# Multimodal graph attention network for COVID-19 outcome prediction

**DOI:** 10.1038/s41598-023-46625-8

**Published:** 2023-11-09

**Authors:** Matthias Keicher, Hendrik Burwinkel, David Bani-Harouni, Magdalini Paschali, Tobias Czempiel, Egon Burian, Marcus R. Makowski, Rickmer Braren, Nassir Navab, Thomas Wendler

**Affiliations:** 1 Computer Aided Medical Procedures and Augmented Reality, School of Computation, Information and Technology, Technical University of Munich, Boltzmannstr. 3, 85748 Garching, Germany; 2 Department of Diagnostic and Interventional Radiology, School of Medicine, Technical University of Munich, Ismaninger Str. 22, 81675 Munich, Germany; 3 Department of Diagnostic and Interventional Neuroradiology, School of Medicine, Technical University of Munich, Ismaninger Str. 22, 81675 Munich, Germany; 4 Department of Radiology, Stanford University School of Medicine, Stanford, CA 94304 USA; 5 Department of Diagnostic and Interventional Radiology and Neuroradiology, Clinical Computational Medical Imaging Research, University Hospital Augsburg, Stenglinstr. 2, 86156 Augsburg, Germany

**Keywords:** Computer science, Medical imaging

## Abstract

When dealing with a newly emerging disease such as COVID-19, the impact of patient- and disease-specific factors (e.g., body weight or known co-morbidities) on the immediate course of the disease is largely unknown. An accurate prediction of the most likely individual disease progression can improve the planning of limited resources and finding the optimal treatment for patients. In the case of COVID-19, the need for intensive care unit (ICU) admission of pneumonia patients can often only be determined on short notice by acute indicators such as vital signs (e.g., breathing rate, blood oxygen levels), whereas statistical analysis and decision support systems that integrate all of the available data could enable an earlier prognosis. To this end, we propose a holistic, multimodal graph-based approach combining imaging and non-imaging information. Specifically, we introduce a multimodal similarity metric to build a population graph that shows a clustering of patients. For each patient in the graph, we extract radiomic features from a segmentation network that also serves as a latent image feature encoder. Together with clinical patient data like vital signs, demographics, and lab results, these modalities are combined into a multimodal representation of each patient. This feature extraction is trained end-to-end with an image-based Graph Attention Network to process the population graph and predict the COVID-19 patient outcomes: admission to ICU, need for ventilation, and mortality. To combine multiple modalities, radiomic features are extracted from chest CTs using a segmentation neural network. Results on a dataset collected in Klinikum rechts der Isar in Munich, Germany and the publicly available iCTCF dataset show that our approach outperforms single modality and non-graph baselines. Moreover, our clustering and graph attention increases understanding of the patient relationships within the population graph and provides insight into the network’s decision-making process.

## Introduction

Reflecting on the coronavirus disease 2019 (COVID-19) pandemic^1^, the first wave, in particular, brought unprecedented challenges to the healthcare system. The exponential surge in cases overwhelmed intensive care units (ICUs), presenting scenes that had never been witnessed in the age of modern medicine^2,3^. During such a state of emergency, optimizing the allocation of hospital resources, e.g., ICU beds, mechanical ventilators, or personnel, becomes crucial. An essential aspect of effective patient management is correctly assessing treatment necessity and potential outcomes. When there is only a limited understanding of a previously unknown disease paired with highly multimodal data, as in the case of a novel pandemic, performing such an assessment and prediction of patient outcomes is very challenging. The resulting sudden overload of care facilities, in combination with the high complexity of the obtained data structure, motivates the need for assistance systems for fast outcome prediction and triaging based on available patient information. At the start of the COVID-19 pandemic, upon a patient’s hospital admittance, a multitude of parameters—such as sex, age, body weight, symptoms, co-morbidities, blood cell counts, inflammatory parameters, biochemical values, cytokine profiles, among others—were obtained and documented^4^. These parameters—“tabular data” in the following—and radiological images, including radiographs or X-ray computed tomography (CT) images, were available within the first hours after a new patient arrived at the hospital. This deems the two data sources ideal for early triaging and outcome prediction. Traditional disease outcome prognosis performed by clinicians is based, however, also on anamnestic information and clinical experiences. The general logic of a physician’s decision-making process partly relies on the information embedded in similar patients where the outcome and the connection of this information to currently treated patients are known^5^. Such population relationships are particularly useful when little to no disease-specific epidemiological information, as well as deep medical expertise, is available, as might be the case in potential upcoming health crises. Following this method of reasoning, we propose a decision support system that performs multimodal data analysis to create a population graph that clusters patients which is then used in a graph neural network with an attention mechanism to refine the patient outcome prediction by taking into account similar patients. The used similarity metric, attention mechanism, and generated pathology segmentations provide added insight into the decision-making process. This becomes possible as the weighting of clinical features and the most influential patients used in the prediction process can be directly observed. Our contributions are as follows:

- We introduce U-GAT, an end-to-end, graph-based method for leveraging medical images, extracted radiomics, and clinical data for predicting patient outcomes. In this work, we use multimodal data to predict COVID-19 patient outcomes, namely ICU admission, need for ventilation, and mortality. This method is generalizable and can easily be adapted to different types of anatomies, modalities, and clinical tasks.
- Our model uses a multitasking approach, where segmentation and classification are learned simultaneously. A U-Net^6^ is used to segment the healthy and pathological regions of the lung in chest CTs. From these segmentations, we extract scalar values, in the following called “radiomics”, e.g., the percentage of healthy or pathological lung tissue volume, and subsequently perform a joint feature fusion of image, radiomic, and clinical features. This combined feature vector is refined in our Graph Attention Network (GAT)^7^ by leveraging similar patients to perform the final outcome prediction.
- We present an interpretable, multimodal patient similarity metric for graph construction and effective batch selection.
- We introduce a novel equidistant image sampling method allowing for end-to-end training of volumetric image feature extraction in a graph convolutional setting with multiple patients per batch graph. At test time, we make use of all available slices.
- We thoroughly evaluate our novel approach on a newly acquired dataset collected in Klinikum rechts der Isar in Munich during the first COVID-19 wave of 2020 as well as an external and publicly available dataset and showcase our model’s ability to predict patient-specific disease outcomes. The dataset from Klinikum rechts der Isar contains expert annotations of a diverse range of COVID-19 pathologies and is available for research purposes upon request.
- While we validate our method on COVID-19, it is disease-agnostic and the insights about modeling multimodal data without prior experience with patient trajectories can be easily adapted to new contexts of novel disease outbreaks.

**Figure 1:**
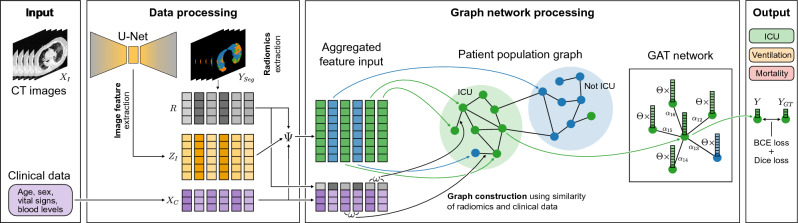
U-GAT is an end-to-end model, integrating learned image and radiomic features ($Z_I$ and *R*) with clinical metadata $X_C$-such as age, sex, vital signs, and blood levels-for disease outcome prediction. Disease-affected area segmentation $Y_{Seg}$ in CT images $X_I$ aids in extracting radiomic features *R* and regularizes image feature $Z_I$ extraction. These features coalesce into a multimodal vector via function $\Psi$. Test patients cluster with training patients in a graph based on radiomic and clinical data feature distance $\omega$. A Graph Attention Network (GAT) then refines the features to predict the most probable outcome *Y*, utilizing learned linear transformation $\Theta$ and patient attention coefficients $\alpha _{ij}$. Comparison to outcome ground truth $Y_{GT}$ is facilitated by binary cross-entropy (BCE), while the Dice loss aids in the auxiliary segmentation task with manual ground truth. In the COVID-19 context, we segment lung CT image pathologies and predict patient ICU admission, ventilation need, and survival for the KRI dataset, and severity for the iCTCF dataset (not shown here).

## Related work

### Fusing imaging and tabular data

Within the field of multi-modal learning, within recent years, different works have been published. One interesting approach to interweave features from multiple modalities was introduced by Perez et al. for visual reasoning tasks^8^. A Feature-wise Linear Modulation (FiLM) layer affinely transformed the output of a Convolutional Neural Network (CNN) with a learned scaling and shifting factor using the text of the input question. Dynamic Affine Feature Map Transform (DAFT)^9^ extended FiLM to combine the features of 3D brain T1-weighted MRI scans and non-imaging biomarkers for Alzheimer’s prediction. DAFT affinely transformed the imaging features extracted by a 3D Fully CNN by a learned scaling and shifting factor using nine non-imaging features, such as age, sex, and genetic factors. A multi-headed cross-attention block has been recently proposed to fuse imaging and tabular data for skin lesion classification using a transformer architecture^10^ showing marginal improvement over joint fusion.

Taleb et al.^11^ introduced ContIG, a self-supervised pre-training approach trained on 500k individuals from the UK Biobank^12^ combining retinal fundus images with genetic information tested on different classification and segmentation downstream tasks. A contrastive loss based on cosine similarity was utilized to decrease the distance of the embeddings of the multimodal features of one patient. Moreover, Duanmu et al.^13^ combined breast MRI scans and clinical biomarkers to predict chemotherapy response. A network trained on the non-imaging data learned scalar weights that were multiplied with the intermediate results from the imaging network to generate feature maps containing interactive information between imaging and tabular data.

Inspired by the holistic decision-making approach taken by experienced physicians and medical boards, which involves integrating knowledge from diverse fields of expertise^14^, there is a growing interest in developing similar machine learning systems. Huang et al.^14^ outlined three methods for integrating features in deep learning models for radiology: merging extracted image features with non-imaging features (early fusion), combining features with a joint end-to-end (image) feature extraction (joint fusion), and consolidating predictions made by independent models (late fusion). Our method employs joint fusion. In contrast to early and late fusion, joint fusion processes the different modalities separately but integrates them during intermediate stages, allowing for inter-modal interactions and joint model training. Backpropagating the loss function to the feature extraction allows the model to optimize the feature extraction based on the final output or prediction error, ensuring a more synergistic learning process. In the following, we review the fusion methods in the context of COVID-19.

#### Early fusion

For the COVID-19 detection and the prediction of patient outcome, most of the proposed methods integrating both imaging and non-imaging data apply early fusion of features^4,15–20^. Chassagnon et al.^21^ demonstrated the importance of combining a wide range of non-imaging and extracted imaging features for the outcome prognosis of COVID-19 patients in an ensemble of machine-learning models. Shiri et al.^22^ achieved the best results in COVID-19 survival prediction by combining lesion-specific radiomics and clinical data. Gong et al.^23^ improved the results for predicting severe COVID-19 outcomes by adding blood values to other clinical features and extracted radiomics.

#### Late fusion

Applying late fusion with penalized logistic regression, Ning et al.^24^ reported an improvement in both COVID-19 severity and mortality outcome prediction compared to the stand-alone lung CT CNN and non-imaging Multilayer Perceptron (MLP) models. Tariq et al.^25^ explored different fusion methods for predicting the need for hospitalization of COVID-19 patients and found the early fusion of different electronic medical record features to work best for this task.

#### Joint fusion

To the best of our knowledge, we are the first to propose a joint fusion method combining imaging and non-imaging data to predict ICU admission, ventilation, and mortality, or severity, depending on the dataset used.

### Graph convolutional networks for medical applications

Previous studies have showcased the potential of Graph Convolutional Networks (GCNs) in medical applications, particularly in optimizing the processing of medical image information. Parisot et al.^26^ pioneered using GCNs on population graphs to improve Alzheimer’s and Autism Spectrum Disorder prediction. They also demonstrated that varying the patient information included in the graph setup significantly affects network performance^5^. Later works sought to diminish performance dependencies on graph generation, with Anirudh et al.^27^ suggesting a bootstrapping strategy and ensemble learning for GCNs. Cosmo et al.^28^ introduced a self-learning method for graph construction, integrating both imaging and non-imaging data for optimized GCN learning behavior. Further, GCNs have also been employed in medical image segmentation^29–32^ and Graph Attention Networks (GATs)^7^ have been utilized for patient diagnosis^33,34^.

The aforementioned works leveraged already extracted image features. However, Burwinkel et al.^35^ proposed a methodology that used GCNs on image data directly. They showed that end-to-end processing of imaging and clinical data within a GCN can improve performance due to optimized feature learning. At the same time, the proposed approach allowed for more effective usage of inter-class connections within the graph. We will expand upon this concept within our developed methodology and explain the implications in detail in section “Method”.

### GCNs for COVID-19

In the context of COVID-19 diagnosis, GCNs have mainly been adapted for disease detection. Wang et al.^36^ and Yu et al.^37^ built graphs based on the similarity of extracted CT image features and classified the nodes for the presence of infiltrates. In addition to image features, Song et al.^38^ and Liang et al.^39^ used the acquisition site along with other features to improve COVID-19 detection. Instead of modeling a patient population, Saha et al.^40^ converted edges detected in chest CT and X-ray images to graphs and leveraged these for detecting COVID-19. Huang et al.^41^ used GCNs to refine the segmentation of COVID-19 infections. Finally, Di et al.^42^ learned an uncertainty-vertex hypergraph to distinguish between community-acquired pneumonia and COVID-19. To the best of our knowledge, we propose the first graph-based end-to-end patient outcome prediction method by leveraging a population graph combining chest CTs and tabular patient data.

### Multitask learning for COVID-19

Recent works^4,22,43,44^ on the radiological assessment of COVID-19 patients have shown a high correlation between disease burden and patient outcome, e.g., the probability of ICU admission. Several deep learning methods have been proposed to exploit this correlation with multitasking approaches^45–47^. The majority of the proposed multitask methods focus on the joint detection of COVID-19 infection and the binary segmentation of related pathologies in lung CT images^48–52^. Concerning COVID-19 patient outcome prediction, another set of works applied to multitask learning on the joint estimation of the severity of COVID-19 and various classification and segmentation tasks^53,54^. Similar to our approach, Nappi et al.^55^ used bottleneck features of a pretrained U-Net to predict COVID-19 progression and mortality. However, they did not optimize end-to-end, incorporate clinical patient data, or utilize a graph-based approach for the classification.

## Method

Our proposed method provides an effective way to process multimodal patient information such as CT images $X_I$ combined with clinical data $X_C$ for disease outcome prediction of patients, as shown in Fig. 1. For a COVID-19 patient admitted to the hospital, the three outcomes we predict are the need for ICU admission, the need for mechanical ventilation, and the survival of the patient (for our in-house dataset), while we predict severity for the iCTCF dataset. Additionally, we use the segmentation of COVID-19 pathologies as an auxiliary target to improve the training. From the segmentation output, we calculate radiomic features *R* that represent the relative burden of the lung for each pathology class. To effectively incorporate the different modalities, we introduce a new framework that combines the segmentation capabilities of U-Net with the analytic strengths of GCNs. This network uses a population graph constructed with the similarity of clinical patient data $X_C$ and radiomic features *R* to refine the image features of each patient. The proposed method operates end-to-end to perform an ideal combination of image feature representation learning, U-Net image segmentation, and graph data processing. The graph is pre-computed before training, and at test time, patients are dynamically connected to the graph of patients in the training set to ensure no data leaking during training and allow for usage flexibility in a clinical setting.

### Graph-based image processing

To allow for inference on unseen data samples, we employ spatial graph convolutions. Compared to spectral methods, this approach allows an extension to unseen samples, not requiring retraining for every new patient. As explained in section “Fusing imaging and tabular data”, combining image data $X_I$ with other modalities is essential for a holistic patient outcome prediction. For GCNs, image-based information is usually first extracted either manually or with a pretrained CNN. These extracted image features are then, in a second step, processed within the graph network. While this strategy lessens the memory demands of imaging data, it precludes the possibility of end-to-end optimization. Burwinkel et al.^35^ showed that the image feature extraction process can potentially benefit from an underlying graph structure through an end-to-end feature extraction with a graph neural network since relevant graph information can backpropagate into the learned extraction process. We leverage this concept for the processing of the provided CT image information. Every CT image $x_{I,i}$ is processed by a U-Net to perform segmentation on the individual image slices. The calculated bottleneck feature maps of the U-Net are extracted (description in section “Segmentation and image feature extraction”) and processed to receive a corresponding representation $z_{I,i}$, usable within the graph neural network.

#### Equidistant subsampling

Utilizing GCNs for end-to-end feature extraction from high-resolution 3D images presents a major challenge due to high memory demands, which restricts the number of patient instances per batch. However, GCNs necessitate diversity in a single batch for effective feature aggregation. To accommodate larger batches, we suggest equidistant subsampling of *S* slices per volume along the axial view during training. If the main axis length is *Z*, each volume is divided into $\lfloor Z/S \rfloor$ stacks of *S* slices, omitting $(Z\mod S)/2$ slices on both sides. This strategy not only enhances the likelihood of detecting disease-impacted areas but also mitigates overfitting by distributing scarce 3D volume data into multiple patient samples. At test time, the complete stack of slices is used, encompassing the entire 3D volume.

#### Graph construction method

**Figure 2:**
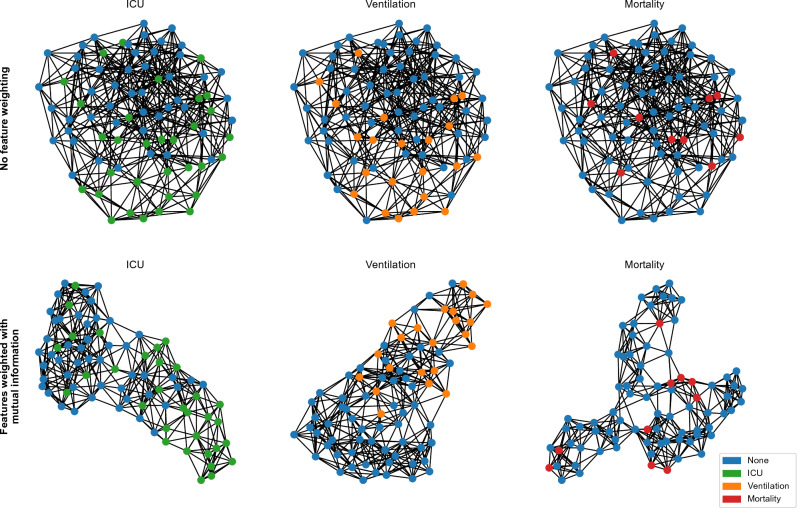
The initial patient clustering, visualized for the KRI dataset, is based on clinical and radiomic feature similarity. The top row displays graphs created by linking each node to its seven nearest neighbors based on Euclidean distance. To optimize this graph construction for the task at hand, we propose feature weighting in the distance calculation, informed by its task-specific mutual information^56^ of features (bottom row). This prioritizes essential features in clustering and tailors the graph for specific tasks without needing feature selection or prior knowledge.

We define a binary, directed graph *G*(*V*, *E*) with vertices *V* and connecting edges *E*. Every vertex $v_i \in V$ corresponds to a stack of CT images $x_{I,i} \in X_I$ (sampling process described in section “Graph-based image processing”), a vector of radiomics features $r_i \in R$ (extraction process described in detail in section “Segmentation and image feature extraction”) and clinical data $x_{C,i} \in X_C$. For building the graph we concatenate the clinical data $X_C$ and radiomics features *R* into one tabular feature and calculate the distance $\omega$ between two vertices based on these features. Each vertex $v_i$ is connected with its *k* nearest neighbors. As an alternative to feature selection, we propose to weight each feature based on a statistical analysis of the training data. Statistically important features should therefore have a bigger influence on the distance and similarity calculation. Possible weightings include correlation coefficients, e.g. the Pearson correlation for continuous features, or estimated mutual information^56^ between the input features and the target labels like $Y_{\text {ICU}}$ calculated on the training set. The motivation to use mutual information is to discover non-linear associations between the features and predicted labels, in addition to linear relationships. All distances are calculated on the z-scores normalized features. In Fig. 2, the k-nearest neighbors (KNN) graphs for one training set are visualized with and without weighting of the distance with mutual information.

### Segmentation and image feature extraction

The proposed method is built on a joint image feature extraction and segmentation backbone. For this, any encoder-decoder-based architecture with a compressed bottleneck representation and segmentation output can be used. As described in more detail in section “Experiments”, we choose the original 2D U-Net architecture^6^ with small adaptions for our experiments. The *S* equidistant slices forming an input image $x_{I, i}$ (see section “Graph-based image processing”) are processed as a batch in parallel. Hence, for each slice, a 2D segmentation of the healthy lung and pathologies is generated. The image representation used for the classification task is extracted with a global average pooling of the two-dimensional bottleneck features of each slice, reducing the bottleneck size $c\times d_1 \times d_2$ with the number of channels *c* and the spatial dimensions $d_1$ and $d_2$ to a vector with the length of *c* per slice. The resulting *S* slice-wise image representations are then transformed into a single patient-wise representation. To achieve this, the slice features are aggregated by taking the element-wise maximum along the stacking dimension resulting in a single vector with size *c*. This vector is then passed through a final fully connected layer followed by a leaky ReLU activation to obtain the latent image representation $z_{I,i} \in Z_{I}$. Based on the improved performance reported by Goncharov et al.^54^ using the final feature map of the U-Net instead of the bottleneck, we evaluated this approach, but initial results showed a substantial drop in performance which is why we did not investigate this concept any further.

#### Extraction of radiomic features

Inspired by Burian et al.^4^, the clinical data is complemented with radiomics features *R* that are automatically extracted from the segmentation output $Y_{\text {Seg}}$. In addition to being more robust to overfitting than extracted image features, this improves the interpretability of the network by providing intermediate results that can easily be verified by visualizing the segmentation output. For instance, in the case of COVID-19-related tasks, one can use quantifications of COVID-19 pathologies in the segmented lung.

### Multimodal feature fusion

Our methodology harnesses the multimodal data in a two-fold manner. On the one hand, radiomics extracted from the segmentation output and clinical patient parameters are employed to form the patient population graph. On the other hand, we synergistically fuse latent image features, extracted radiomics, and clinical data into the node features of this graph. This integrated representation encapsulates all salient attributes of a patient, providing a comprehensive patient characterization for subsequent processing. The three input sources provided by the image data $x_{I,i} \in X_I$ and resulting extracted features $z_{I,i}$, extracted radiomics features $r_i \in R$ and clinical data $x_{C,i} \in X_C$ constitute three separate modalities used within the graph network to perform the classification task for an individual patient node $v_i$ within the graph. Especially the clinical data $X_C$ can provide valuable orthogonal information to the imaging-based other two contributions. We have incorporated the latent bottleneck features $z_{I,i}$ of the U-Net to allow for end-to-end feature optimization, facilitating an image feature extraction beyond hand-crafted radiomics. To assure that the influence of every modality is equally considered during processing, we are using a linear transformation on every modality to receive a feature representation of equal size. These representations are then processed within an aggregation function $\Psi$ to receive the corresponding fused representation $z_{f,i}$ used within the graph network:

1 $$\begin{aligned} z_{f,i} = \Psi \left( \sigma \left( \Theta _I z_{I,i}\right) , \sigma \left( \Theta _R r_i\right) , \sigma \left( \Theta _C x_{C,i}\right) \right) ~, \end{aligned}$$

where $\sigma$ is a non-linear activation function and $\Theta _I \in {\mathbb {R}}^{F_I \times F_f}$, $\Theta _R \in {\mathbb {R}}^{F_R \times F_f}$, $\Theta _C \in {\mathbb {R}}^{F_C \times F_f}$ are learnable linear transformations, which map the incoming feature dimension onto dimension $F_f$. Possible approaches for $\Psi$ are concatenation, averaging, pooling, or attention mechanisms. Concatenation was experimentally chosen for our proposed method as discussed later in section “Experiments”.

### Classification of patient outcome

The graph processing of our proposed method is based on graph attention layers (GAT)^7^. They combine effective processing of the provided neighborhood with the possibility for direct inference on new unseen data samples while maintaining filter localization and low computational complexity. The attention-based graph processing allows us to incorporate the clinical patient data $X_C$ effectively into the learning process by basing the graph construction on the similarity of tabular features and creating *N*(*i*) for every $z_{f,i}$. Further, the attention mechanism allows for an intelligent learned weighting of the neighbors. Now, a transformation of representation $z_{f,i}$ does not only rely on the representation itself but receives weighted contributions from all $z_{f,j} \in N(i)$. This process has the potential to stabilize the prediction for patients with an uncharacteristic initial representation of its corresponding class, but which is localized within the correct data cluster.

**Table 1:** Backbones and classifiers used for evaluation with the respective features for patients and the distance metric (similarity).

Architecture	Multitasking	Multimodal	Patient modalities			Patient similarity	
			Images	Radiomics	Clinical	Radiomics	Clinical
MLP-Clinical	–	–	–	–	$\checkmark$	–	–
RF-Clinical	–	–	–	–	$\checkmark$	–	–
ResNet18	–	–	$\checkmark$	–	–	–	–
ResNet18-GAT	–	$\checkmark$	$\checkmark$	–	$\checkmark$	–	$\checkmark$
U-Net*+RF	–	$\checkmark$	–	$\checkmark$	$\checkmark$	–	–
U-Net*+KNN	–	$\checkmark$	–	$\checkmark$	$\checkmark$	$\checkmark$	$\checkmark$
U-Net*+MLP	–	$\checkmark$	$\checkmark$	$\checkmark$	$\checkmark$	–	–
U-Net*+GraphSAGE	–	$\checkmark$	$\checkmark$	$\checkmark$	$\checkmark$	$\checkmark$	$\checkmark$
U-GAT*	–	$\checkmark$	$\checkmark$	$\checkmark$	$\checkmark$	$\checkmark$	$\checkmark$
U-GAT	$\checkmark$	$\checkmark$	$\checkmark$	$\checkmark$	$\checkmark$	$\checkmark$	$\checkmark$

*Images* describes the latent image features extracted with an image encoder. *Radiomics* stands for the radiomics extracted from the segmentation networks. *Clinical* data includes vital signs, blood values, and demographic information. We compare U-GAT to other end-to-end trained methods only using clinical data (MLP-Clinical), only using image data (ResNet18), and a GAT with a CNN backbone without an auxiliary segmentation task (ResNet18-GAT). In addition, we compare the performance of different classifiers on the image features extracted from a frozen U-Net, marked with a *, i.e., U-Net*. KNN is a k-nearest neighbors classifier. GraphSAGE is a graph convolutional method without an attention mechanism^57^. Multitasking refers to the joint training of classification and segmentation.

## Experiments

### Datasets

#### KRI dataset

The KRI dataset (“in-house” dataset) consists of 132 COVID-19 patients, expanding on the dataset with 65 patients described in^4^. To assess the patient outcome, different parameters were collected: admission to the ICU, the necessity of mechanical ventilation, and the patient’s survival. These outcomes presented themselves immediately or sometime after general admission to the hospital. The complete dataset is available on request for research purposes in the frame of the BFS project AZ-1429-20C. For each CT volume, the total lung, healthy lung tissue, ground-glass opacifications (GGO), consolidations, and pleural effusions area were annotated by expert radiologists (4-8 years of experience). We combined pleural effusion and consolidation into a single class named “Other pathologies” since distinguishing between the two classes is a highly challenging task, even for senior radiologists^58^ as both have almost the same Hounsfield unit range. Moreover, pleural effusion is only present in the most severe cases in only 1.2% of all available patients. See radiomics statistics for this dataset in the supplementary material.

#### iCTCF dataset

To substantiate the versatility of our method, we have extended our evaluation to a larger and publicly available dataset: the iCTCF dataset^24^ (“external” dataset). It comprises 1,521 patients and includes high-resolution CT images, clinical data, and patient outcomes. The main difference to the KRI annotations is the lack of image annotations of different pathologies in the lung. Since our work focuses on triaging patients infected with COVID-19, we exclude the control group and only predict the outcome severity of PCR-positive COVID-19 patients. This results in 620 patients with mild (Type I) and 274 patients with severe outcomes (Type II)^24^ leading to a total of 894 patients. Since the iCTCF dataset does not contain any annotations of the CT images, we employ a U-Net, pretrained on a diverse dataset^59–61^ of lung CT-slices by Hofmanninger^62^, to generate lung masks and a nnU-Net by Isensee et al.^63^, pretrained on the COVID-19 Lung CT Lesion Segmentation Challenge^64^, to infer the pathology annotation. The radiomic *COVID-19 burden* was extracted using this annotation, resembling the percentage of the lung affected by COVID-19 pathologies.

### Experimental setup

We first evaluate the proposed method on the KRI dataset using a nested 5-fold cross-validation^65^ stratified by the ICU labels. For this, the dataset is split into five equally sized folds, each containing a similar amount of ICU patients. In nested cross-validation, there are outer and inner evaluation loops for testing and validation. In each of the five outer loops, one fold is selected as a test set, and the remaining four folds are used for training and validation. In the four inner loops, three folds are selected for training and one for validation. This is repeated until every combination has been used for testing and validation, resulting in a total of 20 repetitions.

For the experiments presented here, following Burian et al.^4^, the static lung CT images taken at admission were used in combination with the following clinical features and blood test results: age, sex, body temperature, percutaneous oxygen saturation, leukocytes, lymphocytes, C-reactive protein (CRP), creatine, D-Dimer, lactate dehydrogenase (LDH), creatine kinase, troponin T, interleukin 6 (IL-6), thrombocytes. The outcomes included: the need for mechanical ventilation, admission to the ICU, and patient survival (mortality). All three tasks are binary classification tasks. We focus on evaluating the main task of ICU prediction and extend some experiments on ventilation and mortality outcome tasks to explore multitasking and the translation to other tasks. The experiments were conducted with ten equidistant samples ($Z=10$) of the chest CT images, producing nine subvolumes per patient. During training, a random subvolume is chosen for each patient. At validation and test time, the whole patient volume is sampled. Since there is only a single test patient per batch, the pre-computed image features and radiomics of the other patients can be used. During the test phase, a batch graph consists of one test node and 18 neighboring nodes from the training set that serves as a context for this new patient. For all our experiments, we set the modality aggregation function $\psi$ to perform concatenation.

For the iCTCF dataset, following the evaluation of Ning et al.^24^, we split the data in a 10-fold cross-validation regime. In every run, eight folds are used for training and 1 for validation and testing, respectively. Given only a single radiomic of the COVID-19 burden of the lung is available, we concatenate the extracted radiomic with the clinical data and encode this tabular data into a joint embedding vector of size 64 for each patient. Since the dataset contains many features, of which most have only low mutual information with the target outcome, only features with estimated mutual information higher than 0.05 were used for graph construction. All available clinical features were used as patient node features. We stopped training when there was no improvement in the validation classification loss for five epochs.

#### Network parameters and training

We conducted all experiments in PyTorch 1.7.0^66^ and PyTorch Geometric 1.7.0^67^ using the Adam optimizer with a base learning rate of $5 \times 10^{-4}$ and a weight decay of $3\times 10^{-5}$. As the segmentation and image feature extraction backbone, we choose the classical 2D U-Net architecture proposed by Ronneberger et al.^6^ with the following modifications in the double convolution blocks: an added batch normalization layer after each activation for faster convergence and a padding of one pixel in each convolution layer to align input and output image size of the network. The final layer consisted of a one-dimensional convolution to the number of output classes followed by a softmax layer. We used a Dice loss as introduced by Milletari^68^ for segmentation and a binary cross-entropy (BCE) loss for classification. Further training details can be found in the supplementary material. For graph processing, we used a two-layer GAT^7^.

#### Graph construction

We employed the KNN graph construction method introduced in section “Graph-based image processing” using a mutual information weighted distance metric for the following experiments after comparing it to other methods on the validation set. For $\omega$ we chose the weighted Euclidean distance (Minkowski distance of second order, $p=2$). Here, every feature dimension was weighted by its approximate mutual information with the respective outcome label. The mutual information was estimated using the method proposed by Ross et al.^56^ with 3 neighbors averaging the results of 30 repetitions. We compared weighting the KNN with mutual information against weighting with Pearson correlation. To understand the impact of weighting features, we also compared these weighted methods against an unweighted KNN. For the unweighted setup, we evaluated different subsets of manually selected features as can be seen in Table 3. The number of neighbors *k* used for graph construction was set in a hyperparameter search on the validation set.

### Ablative testing and comparison to baselines

To investigate the effect of the different components of our method, we show ablative results on the test set. We mainly evaluate two components: the image and radiomics feature extraction of the U-Net and the GAT classification. The end-to-end U-GAT feature extraction is compared with features extracted from a simple frozen U-Net trained on the same annotations but without any multi-tasking, and the end-to-end image features from a ResNet18 as proposed by He et al.^69^. It is important to note that radiomics were not used in the ResNet18-GAT architecture because ResNet18 does not produce segmentations. To evaluate the contribution of GAT, we compare it with the following classification method alternatives:

- Weighted K-nearest neighbors (KNN): The default scikit-learn weighted k-nearest neighbor classifier using the inverse Euclidean distance of all features as the similarity metric for neighbor selection and for weighting of neighbor labels^70^.
- Multilayer Perceptron (MLP): This classifier is a simple neural network with a hidden layer size of 64 followed by a leaky ReLU activation and a 10% dropout.
- GraphSAGE: replacing the GAT operator with GraphSAGE^57^.

In addition to ablative testing, we compare unimodal vs. multimodal approaches by evaluating the performance of using an MLP classifier using only clinical data or only image features extracted by a ResNet18. An overview of the type of data used in each method is given in Table 1.

#### U-GAT ensemble and comparison with Random Forest

Random Forest is an ensemble method that is an effective classifier for small datasets since they are less prone to overfitting due to the Law of Large Numbers^71^ and provide the additional benefit of interpretability. As discussed in section “Fusing imaging and tabular data”, Burian et al.^4^ and Chao et al.^15^ have successfully deployed Random Forests to use tabular radiomics and clinical data for ICU prediction. In this experiment, we focus on the task of ICU prediction and explore if an ensemble of our proposed model can improve its performance due to increased robustness against overfitting and how it compares to the well-established Random Forest classifier. To form an ensemble we average the predicted probabilities of the 4 models trained on the inner loops of the nested cross-validation and evaluate them on the 5 test sets of the outer loop of the nested cross-validation.

### Metrics for segmentation and classification

As our proposed method follows a multitask approach including the CT segmentation and each of the tasks of ICU, ventilation and mortality prediction individually, the evaluation criteria can be divided into segmentation and classification metrics. To measure the overlap between segmented regions and ground truth, we use the Dice score (DS). The main metrics for evaluating the binary classification performance are average precision (AP) and the area under the receiver operating characteristic curve (AUC), as they are independent of selected classification thresholds. Given that all tasks have a severe class imbalance, the F1 score (F1) has been chosen as the main threshold-dependent metric. In the ensemble experiments, the balanced accuracy score (bACC), sensitivity, and specificity are additionally reported. For all threshold-dependent metrics, the optimal threshold is set using the validation results and maximizing the Youden’s J statistic^72^: ${J={\text {sensitivity}}+{\text {specificity}}-1}.$ The classification metrics are all binary and were calculated using scikit-learn 0.24.1^70^.

**Table 2:** Top 10 features sorted by the mutual information for each task and its Pearson correlation in the KRI dataset.

Task	Feature	Category	Mutual information	Pearson correlation
ICU	Healthy lung (%)	Radiomics	$0.244 \pm 0.052$	$-0.596 \pm 0.033$
ICU	Ground-glass opacity (%)	Radiomics	$0.184 \pm 0.043$	$+0.577 \pm 0.026$
ICU	Other pathologies (%)	Radiomics	$0.144 \pm 0.055$	$+0.471 \pm 0.048$
ICU	C-reactive protein	Clinical	$0.104 \pm 0.038$	$+0.372 \pm 0.071$
ICU	Interleukin 6	Clinical	$0.091 \pm 0.023$	$+0.091 \pm 0.137$
ICU	Age	Clinical	$0.087 \pm 0.031$	$+0.018 \pm 0.062$
ICU	Lymphocytes	Clinical	$0.047 \pm 0.027$	$-0.062 \pm 0.112$
ICU	Temperature	Clinical	$0.043 \pm 0.040$	$-0.016 \pm 0.116$
ICU	Serum creatinine	Clinical	$0.041 \pm 0.045$	$+0.009 \pm 0.125$
ICU	Thrombocytes	Clinical	$0.039 \pm 0.037$	$-0.007 \pm 0.060$
ICU	Creatine kinase (total)	Clinical	$0.037 \pm 0.040$	$+0.113 \pm 0.110$

The average is calculated on the training sets of all repetitions.

**Table 3:** Evaluation of edge features and their weighting used for distance calculation on the validation set of the KRI dataset.

Task	Architecture	Distance features	Distance feature weights	AP	AUC
ICU	U-GAT*	Age, sex	–	$0.512 \pm 0.109$	$0.573 \pm 0.109$
ICU	U-GAT*	Clinical	–	$0.671 \pm 0.152$	$0.720 \pm 0.135$
ICU	U-GAT*	Radiomics	–	$0.670 \pm 0.145$	$0.720 \pm 0.116$
ICU	U-GAT*	All	–	$0.704 \pm 0.080$	$0.733 \pm 0.073$
ICU	U-GAT*	All	Pearson correlation	$0.697 \pm 0.122$	$0.751 \pm 0.088$
ICU	U-GAT*	All	Mutual information	${\textbf {0.722}} \pm {\textbf {0.096}}$	${\textbf {0.757}} \pm {\textbf {0.142}}$

Highest values are in bold.

## Results and discussion

### Population graph construction

In the first phase of experiments on our KRI dataset, we optimized the population graph construction method. This involved evaluating various feature selections and distance weights to improve the KNN-based graph construction. We found that connecting each node with its seven nearest neighbors provided optimal results, based on a hyperparameter search using a simple, unweighted KNN classifier. Two measures - mutual information and Pearson correlation - were used to weight features in the distance calculation of the similarity metric used for KNN neighbor selection. Table 2 shows the top 10 of the average of both measures for the ICU task. While a Pearson correlation $>0.3$ and mutual information $>0.1$ can be observed in the ICU and ventilation tasks for some features, the mortality showed significantly lower values indicating the difficulty of the task at hand (see supplementary material). The percentage of the healthy lung has the highest mutual information for all tasks. The results shown in Table 3 confirmed that our proposed weighting with the mutual information method yielded the best outcomes, particularly for the ICU task, as indicated by an AP of $0.722 \pm 0.096$ and an AUC of $0.757 \pm 0.142$. The comparison with manual feature selection, e.g., only using clinical data, showed that using all available features is most effective, but mutual information estimation can further help identify the most relevant features and give them a higher weight in the similarity metric. The external dataset confirmed the feature importance of radiomic data. Here, the COVID-19 burden has the highest mutual information with the severity labels (see supplementary material). A key benefit of using a weighted distance for KNN graph construction is that the graph can adapt to each task without prior knowledge. Fig. 2 shows the graph for each task on the KRI dataset with and without weighting the distance measure with mutual information. Besides improving classification, an effective similarity measure can be used to identify relevant patients that have been treated in the past and support the decision-making process of physicians by enabling them to analyze the disease progression of similar patients.

### U-GAT evaluation

**Table 4:** Ablative testing and comparison with an MLP only using clinical data and a ResNet18 only using image data as input on all tasks.

Dataset	Task	Architecture	AP	AUC	F1
KRI	ICU	MLP-Clinical	$0.577 \pm 0.109^\dagger$	$0.654 \pm 0.104^\dagger$	$0.560 \pm 0.107^\dagger$
KRI	ICU	ResNet18	$0.670 \pm 0.097$	$0.716 \pm 0.077$	$0.560 \pm 0.084^\dagger$
KRI	ICU	U-Net*+KNN	$0.632 \pm 0.113$	$0.677 \pm 0.112$	$0.519 \pm 0.131^\dagger$
KRI	ICU	U-Net*+MLP	$0.615 \pm 0.127^\dagger$	$0.687 \pm 0.128$	$0.612 \pm 0.085$
KRI	ICU	U-Net*+GraphSAGE	$0.628 \pm 0.114^\dagger$	$0.690 \pm 0.107^\dagger$	$0.574 \pm 0.085^\dagger$
KRI	ICU	ResNet18-GAT	$0.637 \pm 0.165$	$0.678 \pm 0.160$	$0.595 \pm 0.084^\dagger$
KRI	ICU	U-GAT*	$0.672 \pm 0.129$	$0.725 \pm 0.107$	$0.651 \pm 0.104$
KRI	ICU + Seg.	U-GAT	${\textbf {0.699}} \pm {\textbf {0.149}}$	${\textbf {0.743}} \pm {\textbf {0.103}}$	${\textbf {0.661}} \pm {\textbf {0.084}}$

KRI	Ventilation	MLP-Clinical	$0.527 \pm 0.167$	$0.692 \pm 0.109^\dagger$	$0.475 \pm 0.188$
KRI	Ventilation	ResNet18	$0.573 \pm 0.127$	$0.715 \pm 0.086^\dagger$	$0.390 \pm 0.160^\dagger$
KRI	Ventilation	U-Net*+KNN	$0.527 \pm 0.180^\dagger$	$0.674 \pm 0.112^\dagger$	$0.368 \pm 0.192^\dagger$
KRI	Ventilation	U-Net*+MLP	$0.587 \pm 0.183$	$0.741 \pm 0.119$	$0.488 \pm 0.134$
KRI	Ventilation	U-Net*+GraphSAGE	$0.603 \pm 0.151$	$0.758 \pm 0.109$	$0.481 \pm 0.205$
KRI	Ventilation	ResNet18-GAT	$0.570 \pm 0.152$	$0.689 \pm 0.152^\dagger$	$0.423 \pm 0.178^\dagger$
KRI	Ventilation	U-GAT*	$0.618 \pm 0.137$	${\textbf {0.788}} \pm {\textbf {0.106}}$	${\textbf {0.592}} \pm {\textbf {0.130}}$
KRI	Vent. + Seg.	U-GAT	${\textbf {0.644}} \pm {\textbf {0.142}}$	${\textbf {0.788}} \pm {\textbf {0.112}}$	$0.539 \pm 0.179$

KRI	Mortality	MLP-Clinical	$0.261 \pm 0.135$	$0.544 \pm 0.134$	$0.224 \pm 0.152$
KRI	Mortality	ResNet18	$0.210 \pm 0.116^\dagger$	$0.461 \pm 0.155^\dagger$	$0.155 \pm 0.138$
KRI	Mortality	U-Net*+KNN	$0.257 \pm 0.137$	$0.512 \pm 0.166$	$0.184 \pm 0.147$
KRI	Mortality	U-Net*+MLP	$0.252 \pm 0.157$	$0.502 \pm 0.191$	$0.190 \pm 0.157$
KRI	Mortality	U-Net*+GraphSAGE	$0.270 \pm 0.143$	$0.568 \pm 0.180$	$0.236 \pm 0.163$
KRI	Mortality	ResNet18-GAT	$0.247 \pm 0.151$	$0.520 \pm 0.156$	$0.184 \pm 0.157$
KRI	Mortality	U-GAT*	$0.271 \pm 0.137$	$0.549 \pm 0.188$	${\textbf {0.230}} \pm {\textbf {0.172}}$
KRI	Mort. + Seg.	U-GAT	${\textbf {0.287}} \pm {\textbf {0.186}}$	${\textbf {0.586}} \pm {\textbf {0.187}}$	$0.199 \pm 0.173$

iCTCF	Severity	MLP-Clinical	$0.556 \pm 0.099$	$0.735 \pm 0.068$	${\textbf {0.539}} \pm {\textbf {0.064}}$
iCTCF	Severity	ResNet18	$0.525 \pm 0.140$	$0.739 \pm 0.083$	$0.513 \pm 0.102$
iCTCF	Severity	U-Net*+KNN	$0.456 \pm 0.070^\dagger$	$0.705 \pm 0.060$	$0.318 \pm 0.129^\dagger$
iCTCF	Severity	U-GAT*	$0.558 \pm 0.102$	$0.740 \pm 0.096$	$0.505 \pm 0.114$
iCTCF	Severity	U-GAT	${\textbf {0.593}} \pm {\textbf {0.106}}$	${\textbf {0.763}} \pm {\textbf {0.085}}$	$0.521 \pm 0.109$

Highest values per task are in bold.

U-GAT* refers to the proposed method using image and radiomic features extracted from frozen U-Net trained on the same annotations as the end-to-end U-GAT. Values marked with $\dagger$ indicate statistical significance with p < 0.05 based on the Wilcoxon’s rank test comparing the proposed method with every other baseline.

**Table 5:** Comparative analysis of ICU outcome prediction on the KRI dataset: U-GAT vs its cross-validation ensemble, a random forest model using only clinical data, and another random forest model incorporating all available tabular data, including radiomics extracted with a pretrained U-Net.

Architecture	AP	AUC	bACC	F1	Sens.	Spec.
RF-Clinical	$0.635 \pm 0.098$	$0.707 \pm 0.086$	$0.624 \pm 0.056$	$0.519 \pm 0.070$	$0.475 \pm 0.131$	$0.773 \pm 0.175$
U-Net*+RF	$0.729 \pm 0.089$	${\textbf {0.774}} \pm {\textbf { 0.057}}$	$0.716 \pm 0.075$	$0.649 \pm 0.011$	$0.651 \pm 0.177$	${\textbf {0.781}} \pm {\textbf {0.166}}$
U-GAT ensemble	${\textbf {0.745}} \pm {\textbf {0.137}}$	$0.770 \pm 0.098$	${\textbf {0.735}} \pm {\textbf {0.111}}$	${\textbf {0.700}} \pm {\textbf {0.114}}$	${\textbf {0.736}} \pm {\textbf {0.067}}$	$0.734 \pm 0.174$

Highest values are in bold.

In the next set of experiments shown in Table 4, we evaluate the different components of the proposed method and compare the results to baseline methods. Our multimodal method outperforms the unimodal MLP, limited to only clinical data as input. The same picture presents when limiting the model to solely use imaging data, as is the case for the ResNet18 method. Here, again our proposed methods outperform ResNet18 on all tasks. These experiments showcase the benefit of a multimodal approach. U-GAT achieves a higher AP than the other methods in all ablations of replacing the U-Net with a ResNet18 and replacing the GAT with an MLP or a GraphSAGE. This shows that leveraging similar patients from the training set is useful for refining the features of test patients. We see similar results on the external dataset where U-GAT has a higher AP of $0.593 \pm 0.106$ than the single modality models MLP and ResNet18 with $0.556 \pm 0.099$ and $0.525 \pm 0.140$, respectively, highlighting the advantage of multimodal learning.

The results of joint end-to-end training of the segmentation and classification task seem to improve the AP slightly for all tasks on both datasets. While the average Dice score is lower in all multitask setups than in the segmentation single-task setup (see supplementary material), this makes the segmentation task a suitable auxiliary task to improve classification results. On the KRI dataset, both the ICU and ventilation predictions reached the highest AP of $0.699\pm 0.149$ and $0.644\pm 0.142$, respectively, when multitasking with segmentation. The mortality task generally achieves worse results. One main explanation for this effect is the immense data imbalance that is present for the mortality task, with only 19 out of 132 positive samples. Additionally, we observe low mutual information of the radiomics and clinical features with the mortality outcome (supplementary material, Table S5). This indicates that the features at hand might not be sufficiently predictive for this specific task. Several relevant clinical aspects closely connected to multiorgan failure, such as heart, kidney and liver parameters, were not available in the datasets. The evaluation on the external dataset shows the same picture where joint end-to-end training of severity classification and pathology segmentation with U-GAT increases the AP from $0.558 \pm 0.102$ to $0.593 \pm 0.106$ compared to U-GAT* that uses segmentations from a frozen U-Net trained on the same annotations.

#### Multitasking evaluation

We conducted additional experiments on the synergistic effects of multitasking segmentation with classification and the concurrent prediction of different patient outcomes since all of these tasks are interdependent. Results detailed in the supplementary material showed that classification can benefit from joint segmentation (Supplementary, Table S7) but mortality prediction was the only task that improved with the simultaneous prediction of all outcomes (supplementary material, Table S8).

#### U-GAT ensemble and comparison with Random Forest

As discussed in section “Ablative testing and comparison to baselines”, we also compare our method against Random Forests used in previous works to perform classification from fused tabular radiomics with clinical data. The comparison, shown in Table 5, illustrates the enhancement in U-GAT’s average precision from $0.699\pm 0.149$ to $0.745 \pm 0.137$, elevating it to marginally outperform the Random Forest, which stands at $0.729 \pm 0.089$. The results indicate that ensembling our method increases the robustness of our method to overfitting, showing comparable results as a Random Forest.

**Figure 3:**
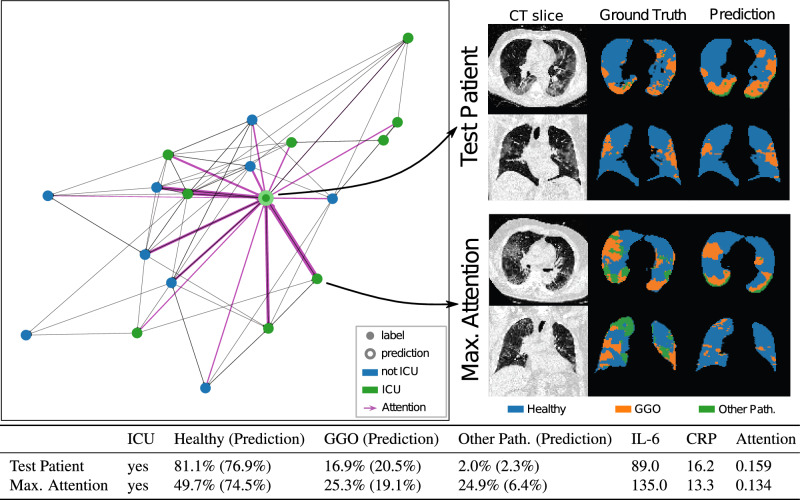
KRI dataset—Left: Batch graph showing the attention scores of a single test patient. The line’s thickness corresponds to the respective neighbors’ attention score after two hops. Right: CT images, segmentation ground truth, and predicted segmentation of a single axial and coronal slice from the test patient and the neighbor with maximum attention. Bottom: Most important features for the test patient and the neighbor with maximum attention. In brackets, the radiomics predicted by the pretrained U-Net are shown.

### Interpretability and inter-patient graph attention

In addition to its performance boost over GraphSAGE, using GAT offers another important advantage. The attention mechanism of our model learns to identify the neighbors in the graph that are the most relevant for the prediction task, providing insight into the decision process of the model. The analysis of attention scores could suggest patients that the model deems relevant for the individual outcome prediction. These connections within the patient population graph can help uncover new information about a disease that is still poorly understood and provide valuable insights to physicians. Combined with the segmentation results, our attention mechanism allows the clinicians to thoroughly evaluate our model output and decision-making process, giving them potentially higher confidence in the prediction. For each of the two GAT layers, the model assigns attention scores to the neighbors of each node in the graph. These scores define how much the node representation after the layer will be based on the representation of its different one-hop neighbors. These attention scores can be thought of as a weighted directed adjacency matrix $A\in [0,1]^{N\times N}$, where *N* is the number of nodes in the batch and all rows in *A* add up to 1. We can multiply the attention matrices of both layers to receive a matrix that shows how the representation of a node is based on its two-hop neighborhood, i.e., all nodes that are at most two edges away. These attention scores are visualized in Fig. 3. Our results on the test patient shown in Fig. 3 highlight that the attention mechanism succeeds in assigning high importance to neighbors of the same class and lower importance to those of the opposite class, thus implicitly refining the neighborhood constructed by the KNN algorithm. Furthermore, we can see that the attention mechanism does not necessarily assign high attention to neighbors that are particularly similar in their radiomic or clinical features. In contrast to a simple KNN classifier, which can only base its prediction on feature similarity, our method evidently can identify the most relevant neighbors that go beyond a simple correlation and are connected through more complex patterns and thus introduces orthogonal information to that embedded in the KNN graph.

### Challenges and future outlook

In a future iteration of our current model, the segmentation of infrequent lung pathologies, such as pleural effusion could be improved along with the prediction of imbalanced outcomes, notably mortality. Our approach to enhance the model involved constructing the population graph based on the mutual information of each feature. This has effectively improved the graph structure, and importantly, the features identified through this method are consistent with established radiological findings. It should be noted, however, that the mutual information displays a pronounced standard deviation and is notably lower for the mortality prediction task, indicating the inherent complexity of this specific prediction task and the potential sparsity of highly informative features given the available parameters in the dataset. In subsequent studies, these areas can be addressed by incorporating more annotated data and expanding the patient cohort, particularly the clinical data.

## Conclusion

In this work, we developed and evaluated a method to effectively leverage multimodal information for the outcome prediction of COVID-19 patients. Here, the said information in the form of CT lung scans, clinical data, and radiomics was incorporated into a graph structure and processed within a GAT to stabilize and support the prediction based on data similarity. With U-GAT, we propose an end-to-end methodology that segments patient pathologies in medical images and uses a combination of imaging and non-imaging data to predict clinical outcomes. We explicitly incorporate automatically extracted lung radiomics in our architecture and demonstrate increased performance. We show that the auxiliary segmentation of COVID-19 pathologies indeed improves outcome prediction. To create the patient population graph, we propose a novel graph construction based on feature weighting utilizing mutual information, effectively clustering relevant patients. Our attention analysis imparts an additional layer of transparency, potentially increasing clinicians’ confidence in our predictive approach. This added clarity can assist in identifying comparable patients from previous cases, thus informing and guiding the treatment trajectory for the current patient under consideration. This study underscores the potential of graph-based, data-driven strategies in improving patient care and decision-making in challenging clinical settings using multiple modalities.

### Supplementary Information

Supplementary Information.

## Data Availability

The iCTCF dataset^24^ is publicly available and can be accessed at https://ngdc.cncb.ac.cn/ictcf/. The complete KRI dataset is available on request for research purposes in the frame of the BFS project AZ-1429-20C.
