# Supplementary material for: Multimodal graph attention network for COVID-19 outcome prediction
Source: Sci Rep. 2023 Nov 9;13:19539. doi: 10.1038/s41598-023-46625-8 (PMC10636061; doi:10.1038/s41598-023-46625-8)
Supplement: Supplementary file 1 — Supplementary Information. [file 41598_2023_46625_MOESM1_ESM.pdf]

Supplementary material

KRI data acquisition

The data was collected retrospectively with the consent of the local institutional review board (ethics approval 111/20 S-KH). All 132 patients (aged 24-99 years, avg. 63 years; 88 males, 44 females) were hospitalized at our institution between April 3rd and September 5th, 2020. For all these patients, COVID-19 was confirmed by the polymerase chain reaction (PCR) test. 53 patients had to be admitted to the ICU for further treatment. Of these, 38 required machine-assisted ventilation, while 19 were deceased.

Patients who were showing at least one of the following symptoms were admitted to the ICU: respiratory rate of over 30 breaths per minute, a peripheral resting state with oxygen saturation less than 93%, invasively measured arterial oxygen tension (PaO2)/inspiratory oxygen fraction (FiO2) of less than 300 mmHg (1 mmHg = 0.133 kPa), respiratory failure requiring mechanical ventilation, cardiovascular shock, and miscellaneous organ failure.

At admission to the hospital, patients presented fever in 66%, coughing in 45%, shortness of breath in 33%, and gastrointestinal symptoms in 15% of the cases, respectively. Percutaneous oxygen saturation was $93.4 \pm 7.1\%$, and the temperature was $37.7 \pm 1.0°C$. Oxygen saturation was significantly different in ICU and non-ICU patients ($90.7 \pm 10.2\%$ vs. $95.0 \pm 3.5\%$), as well as between patients requiring ventilation and not ($89.4 \pm 10.5\%$ vs. $94.8 \pm 4.9\%$). Blood tests were made on admission and can be seen in Tables S2, S3, and S4 with statistics and t-test results.

The non-contrast low-dose lung CT images were acquired at admission using a 256-row multidetector computed tomography (MDCT) scanner (iCT, Philips Healthcare, Best, The Netherlands) in full inspiration with arms elevated.

Table S1. KRI dataset - Radiomics derived from manually annotated CT images at admission for patients. * = significant difference assuming a 5% significance level.

Radiomic	Average	Std. Dev.	Average	Std. Dev.	p
	ICU (n=53)		No ICU (n=79)		
Healthy lung	65.2%	25.9%	92.1%	9.2%	<0.01*
GGO	22.7%	16.4%	6.2%	7.1%	<0.01*
Other pathologies	12.1%	14.0%	1.9%	4.2%	<0.01*
	Ventilation (n=38)		No Ventilation (n=94)		
Healthy lung	61.2%	22.5%	89.4%	16.1%	<0.01*
GGO	25.7%	14.9%	7.7%	10.1%	<0.01*
Other pathologies	13.1%	13.1%	3.2%	7.9%	<0.01*
	Passed (n=19)		Survived (n=113)		
Healthy lung	70.0%	20.5%	83.2%	22.0%	0.02*
GGO	22.0%	17.2%	11.1%	13.2%	<0.01*
Other pathologies	8.0%	9.7%	5.7%	10.8%	0.39

Data processing

KRI data

The CT images were provided with varying sub-millimeter spacing and resampled to an isotropic spacing of 3.6 mm, resulting in an image size of $96 \times 96 \times 96$ voxels. We clipped the Hounsfield in a range of -1024 and 150 for improved contrast of the lung cavity and further applied a patient-level min-max normalization by subtracting the minimum value and dividing by the maximum value of the volume.

All numerical input data, such as clinical data, were z-score normalized by subtracting the mean and dividing by the standard deviation. Missing values were replaced with mean imputation.

iCTCF data

Due to a high variation in the spacing between axial CT slices throughout the dataset, we only resampled the slices within the axial plane to an isotropic spacing of 1.2mm and kept the original number of axial CT slices with an image size of 224×224. All other processing was carried out analogously to the KRI dataset as described in section .

Table S2. KRI dataset - Blood values at admission for the 53 patients who were admitted to the ICU and for the 79 patients who were not. The sum of n differs from our total amount of patients ($n = 132$) due to missing values for some patients. * denotes the significant difference assuming a 5% significance level.

	ICU (n=53)			No ICU (n=79)			
Blood value	Average	Std. Dev.	n	Average	Std. Dev.	n	p
Leukocytes (G/L)	8.4	4.9	53	6.7	4.1	79	0.03*
Lymphocytes (G/L)	19.3	46.9	48	22.6	35.3	75	0.65
Thrombocytes (G/L)	226.6	100.1	53	228.5	116.8	79	0.92
C-reactive protein (CRP. mg/dL)	12.19	9.30	53	6.10	6.26	78	<0.01*
Creatinine (mg/dL)	1.56	1.67	53	4.17	26.50	78	0.48
D-Dimer (μg/mL)	5467	12801	41	1952	5570	67	0.05
Lactate dehydrogenase (LDH. U/L)	468.6	329.5	48	358.4	368.4	75	0.09
Creatinine kinase (U/L)	427.3	1167.2	48	225.3	777.2	74	0.25
Troponine-T (ng/mL)	0.071	0.161	25	0.097	0.323	34	0.71
Interleukin 6 (IL-6. pg/mL)	120.5	117.5	35	104.1	388.7	60	0.81

Table S3. KRI dataset - Blood values at admission for the 38 patients that needed ventilation and for the 94 that did not. The sum of n differs from our total amount of patients ($n = 132$) due to missing values for some patients. * denotes significant difference assuming a 5% significance level.

	Ventilation (n=38)			No Ventilation (n=94)			
Blood value	Average	Std. Dev.	n	Average	Std. Dev.	n	p
Leukocytes (G/L)	7.9	3.7	38	7.2	4.8	94	0.44
Lymphocytes (G/L)	13.1	7.9	35	24.6	46.8	88	0.15
Thrombocytes (G/L)	209.8	102.3	38	235.0	112.7	94	0.23
C-reactive protein (CRP. mg/dL)	13.54	9.80	38	6.53	6.43	93	<0.01*
Creatinine (mg/dL)	1.42	0.66	38	3.81	24.28	93	0.55
D-Dimer (μg/mL)	5622	14484	29	2429	6019	79	0.11
Lactate dehydrogenase (LDH. U/L)	454.1	241.9	36	379.6	393.5	87	0.29
Creatinine kinase (U/L)	533.7	1354.8	35	212.7	718.0	87	0.09
Troponine-T (ng/mL)	0.049	0.089	19	0.104	0.316	40	0.47
Interleukin 6 (IL-6. pg/mL)	138.2	126.4	24	100.7	358.5	71	0.62

Implementation details

U-GAT network architecture

The initial filter size of the convolutions is reduced to 32 instead of 64 to accommodate the small input image size of 96×96 pixels. We train the model with a batch size of 18 patients, each consisting of 10 equidistant slices randomly sampled and the accommodating clinical data. A batch normalization and 10% dropout are applied to the concatenated feature vector Z consisting of image, radiomics, and clinical features before being passed to the classification head. Since it drastically decreased the image segmentation performance, we did not backpropagate the classification loss through extracted radiomics R over the U-Net output. We evaluated concatenation, averaging, and max pooling for the feature fusion Ψ. Following a performance evaluation on the validation set, we received marginally better performance for concatenation without a significant difference compared to the two other approaches. We, therefore, relied on the concatenation approach within our performed experiments.

For the graph-based classification head, we choose a GAT with two layers, five attention heads, and a dropout rate of 10%. Each node feature vector with an input size of 96 gets refined to a feature size of 64 in the first layer and is reduced to a feature size equal to the number of classification labels in the final node classification layer. All binary classification outputs are finally activated with a sigmoid function.

Table S4. KRI dataset - Blood values at admission for the 113 patients who survived and for the 19 who passed. The sum of n differs from our total amount of patients ($n = 132$) due to missing values for some patients. * denotes significant difference assuming a 5% significance level.

	Passed (n=19)			Survived (n=113)			
Blood value	Average	Std. Dev.	n	Average	Std. Dev.	n	p
Leukocytes (G/L)	9.8	6.7	19	7.0	3.9	113	0.01*
Lymphocytes (G/L)	11.3	8.3	17	22.9	42.9	106	0.27
Thrombocytes (G/L)	201.4	99.2	19	232.2	111.6	113	0.26
C-reactive protein (CRP. mg/dL)	11.06	8.98	19	8.14	7.99	112	0.15
Creatinine (mg/dL)	1.57	0.79	19	3.37	22.13	112	0.72
D-Dimer (μg/mL)	6388	12076	13	2862	8644	95	0.19
Lactate dehydrogenase (LDH. U/L)	607.3	500.1	17	368.4	318.7	106	0.01*
Creatinine kinase (U/L)	843.2	1878.8	17	217.6	673.0	105	0.01*
Troponine-T (ng/mL)	0.120	0.216	13	0.077	0.279	46	0.61
Interleukin 6 (IL-6. pg/mL)	143.7	181.9	11	105.8	330.1	84	0.71

Graph construction

Inspired by (1), the similarity $\mathrm{Sim}(u,v)$ between the two nodes u,v is derived from the distance by applying a radial basis function kernel with the mean distance μ calculated on the training set:

$$\mathrm{Sim}(u,v) = \exp\left(-\frac{\omega(u,v)}{2\mu^2}\right). \quad (1)$$

Training details

We conducted all experiments in PyTorch 1.7.0 (2) and PyTorch Geometric 1.7.0 (3) using the Adam optimizer with a base learning rate of 5×10^{-4} and a weight decay of 3×10^{-5}. For the KRI dataset, all models were trained on an NVIDIA Titan V 12GB GPU using Polyaxon. We define an epoch as 80 patients and train the model for a minimum of 25 epochs in the end-to-end case and for a minimum of 5 epochs when using a pretrained U-Net. Experiments using a pretrained U-Net are indicated with an *, i.e., U-Net*. After the minimum amount of epochs, we stop the training if the validation loss has not improved for five epochs. In the end-to-end experiments with joint segmentation and classification, we employ a pretraining schedule since it improved both segmentation and classification results in our preliminary experiments. Here the classification loss was set to zero for the first 20 epochs, and the segmentation loss was only trained on the lung masks for the first ten epochs and then for another ten epochs on all segmentation labels.

The scikit-learn library 0.24.1 (4) was used for estimating the mutual information and KNN graph construction. SciPy 1.6.2 (5) was used for correlation calculation, and NumPy 1.18.2 (6) for all distance calculations.

The Random Forest was implemented using scikit-learn 0.24.1 (4) with default parameters.

Data augmentation

To prevent overfitting on the limited training data, the following augmentation methods were randomly applied to each volume during data loading: intensity scaling with a factor of up to ± 0.15, rotation up to $\pm 10°$ around all axes, and an isotropic scaling with a factor between 0.9 and 1.2. To always have the same input size, bigger volumes were cropped randomly and padded with zeros if needed. During validation and testing, the volumes were cropped centrally.

Table S5. KRI dataset - Top 10 features sorted by the mutual information for each task and its Pearson correlation. The average is calculated on the training sets of all repetitions. In the multilabel setup, the mutual information with the ordinal regression of outcome severity is estimated for each feature.

Task	Feature	Category	Mutual information	Pearson correlation
ICU	Healthy lung (%)	Radiomics	0.244 ± 0.052	-0.596 ± 0.033
ICU	Ground-glass opacity (%)	Radiomics	0.184 ± 0.043	$+0.577 \pm 0.026$
ICU	Other pathologies (%)	Radiomics	0.144 ± 0.055	$+0.471 \pm 0.048$
ICU	C-reactive protein	Clinical	0.104 ± 0.038	$+0.372 \pm 0.071$
ICU	Interleukin 6	Clinical	0.091 ± 0.023	$+0.091 \pm 0.137$
ICU	Age	Clinical	0.087 ± 0.031	$+0.018 \pm 0.062$
ICU	Lymphocytes	Clinical	0.047 ± 0.027	-0.062 ± 0.112
ICU	Temperature	Clinical	0.043 ± 0.040	-0.016 ± 0.116
ICU	Serum creatinine	Clinical	0.041 ± 0.045	$+0.009 \pm 0.125$
ICU	Thrombocytes	Clinical	0.039 ± 0.037	-0.007 ± 0.060
ICU	Creatine kinase (total)	Clinical	0.037 ± 0.040	$+0.113 \pm 0.110$
Ventilation	Healthy lung (%)	Radiomics	0.212 ± 0.033	-0.581 ± 0.030
Ventilation	Ground-glass opacity (%)	Radiomics	0.170 ± 0.022	$+0.585 \pm 0.026$
Ventilation	Other pathologies (%)	Radiomics	0.159 ± 0.055	$+0.428 \pm 0.051$
Ventilation	Interleukin 6	Clinical	0.114 ± 0.048	$+0.109 \pm 0.130$
Ventilation	C-reactive protein	Clinical	0.082 ± 0.047	$+0.395 \pm 0.070$
Ventilation	Temperature	Clinical	0.082 ± 0.044	$+0.031 \pm 0.118$
Ventilation	Age	Clinical	0.059 ± 0.037	$+0.056 \pm 0.053$
Ventilation	Serum creatinine	Clinical	0.055 ± 0.034	-0.020 ± 0.063
Ventilation	Lactate dehydrogenase	Clinical	0.053 ± 0.028	$+0.104 \pm 0.060$
Ventilation	Percutaneous oxygen saturation	Clinical	0.052 ± 0.017	-0.285 ± 0.074
Ventilation	Creatine kinase (total)	Clinical	0.045 ± 0.046	$+0.160 \pm 0.106$
Mortality	Healthy lung (%)	Radiomics	0.061 ± 0.040	-0.210 ± 0.093
Mortality	C-reactive protein	Clinical	0.048 ± 0.034	$+0.126 \pm 0.072$
Mortality	Lymphocytes	Clinical	0.034 ± 0.040	-0.095 ± 0.030
Mortality	Percutaneous oxygen saturation	Clinical	0.033 ± 0.038	-0.023 ± 0.068
Mortality	Interleukin 6	Clinical	0.031 ± 0.013	$+0.068 \pm 0.096$
Mortality	D-dimer	Clinical	0.030 ± 0.023	$+0.122 \pm 0.117$
Mortality	Temperature	Clinical	0.022 ± 0.026	-0.014 ± 0.068
Mortality	Lactate dehydrogenase	Clinical	0.019 ± 0.024	$+0.246 \pm 0.070$
Mortality	Sex	Clinical	0.019 ± 0.010	-0.150 ± 0.041
Mortality	Ground-glass opacity (%)	Radiomics	0.018 ± 0.023	$+0.265 \pm 0.079$
Mortality	Other pathologies (%)	Radiomics	0.016 ± 0.024	$+0.083 \pm 0.100$
Multilabel	Healthy lung (%)	Radiomics	0.274 ± 0.063	-0.548 ± 0.051
Multilabel	Ground-glass opacity (%)	Radiomics	0.190 ± 0.052	$+0.550 \pm 0.042$
Multilabel	Other pathologies (%)	Radiomics	0.173 ± 0.057	$+0.407 \pm 0.066$
Multilabel	Interleukin 6	Clinical	0.105 ± 0.040	$+0.098 \pm 0.133$
Multilabel	Sex	Clinical	0.104 ± 0.110	-0.167 ± 0.052
Multilabel	C-reactive protein	Clinical	0.100 ± 0.043	$+0.352 \pm 0.068$
Multilabel	Lymphocytes	Clinical	0.062 ± 0.044	-0.116 ± 0.046
Multilabel	Age	Clinical	0.057 ± 0.028	$+0.057 \pm 0.062$
Multilabel	Percutaneous oxygen saturation	Clinical	0.047 ± 0.055	-0.233 ± 0.069
Multilabel	Troponin T	Clinical	0.040 ± 0.021	$+0.068 \pm 0.103$
Multilabel	Temperature	Clinical	0.036 ± 0.038	-0.015 ± 0.108

Table S6. iCTCF dataset - Top 10 features sorted by their mutual information with the outcome severity Type I vs. Type II and its Pearson correlation. The average is calculated on the training sets of all ten folds. Overall the mutual information and Pearson correlation are lower than for the tasks of the KRI dataset. The COVID-19 burden radiomic extracted from the U-Net is equivalent to one minus the healthy lung percentage and similarly ranks among the most important features.

Task	Feature	Category	Mutual information	Pearson correlation
iCTCF Severity	Neutrophil percentage (NEP)	Clinical	0.074 ± 0.011	0.315 ± 0.016
iCTCF Severity	COVID-19 burden	Radiomic	0.067 ± 0.011	0.376 ± 0.021
iCTCF Severity	Lymphocyte percentage (LYP)	Clinical	0.066 ± 0.014	-0.293 ± 0.017
iCTCF Severity	Lymphocyte count (LY)	Clinical	0.066 ± 0.018	-0.229 ± 0.029
iCTCF Severity	Prothrombin time (PT)	Clinical	0.045 ± 0.013	0.063 ± 0.011
iCTCF Severity	Calcium (CA)	Clinical	0.043 ± 0.008	-0.242 ± 0.015
iCTCF Severity	D-dimer (DD)	Clinical	0.032 ± 0.008	0.225 ± 0.015
iCTCF Severity	Albumin (ALB)	Clinical	0.032 ± 0.006	-0.269 ± 0.021
iCTCF Severity	Basophil percent (BAP)	Clinical	0.030 ± 0.007	-0.062 ± 0.011
iCTCF Severity	Neutrophil count (NE)	Clinical	0.030 ± 0.016	0.220 ± 0.018

Table S7. Comparison of test set DICE scores of U-Net and a multitasking U-GAT: The joint optimization of segmentation and classification leads to a minor decrease in segmentation metrics. Dice COVID-19 refers to all pathologies reated to COVID-19.

Dataset	Task	Architecture	Segmentation	Classification	Dice lung	Dice COVID-19
KRI	-	U-Net	✓	-	0.867 ± 0.127	0.450 ± 0.232
KRI	ICU	U-GAT	✓	✓	0.862 ± 0.134	0.380 ± 0.238
iCTCF	-	U-Net	✓	-	0.984 ± 0.002	0.738 ± 0.019
iCTCF	Severity	U-GAT	✓	✓	0.970 ± 0.038	0.718 ± 0.027

Table S8. Multitask classification results for pathology segmentation with joint prediction of three patient outcomes: ICU admission, need for ventilation, and mortality. Graphs are constructed using ordinal regression of the severity of the predicted outcomes. Predictions are modeled as a multilabel classification. Notably, only the mortality prediction shows improvement compared to single-task classification with joint segmentation.

Dataset	Task	Architecture	AP	AUC	F1
KRI	ICU	U-GAT	0.649 ± 0.128	0.697 ± 0.116	0.569 ± 0.163
KRI	Ventilation	U-GAT	0.622 ± 0.127	0.774 ± 0.094	0.503 ± 0.188
KRI	Mortality	U-GAT	0.289 ± 0.138	0.620 ± 0.175	0.216 ± 0.174

References

1. Parisot, S. *et al.* Spectral graph convolutions for population-based disease prediction. In *International conference on medical image computing and computer-assisted intervention*, 177–185 (Springer, 2017).

2. Paszke, A. *et al.* Pytorch: An imperative style, high-performance deep learning library. *Adv. neural information processing systems* **32**, 8026–8037 (2019).

3. Fey, M. & Lenssen, J. E. Fast graph representation learning with pytorch geometric. *arXiv preprint arXiv:1903.02428* (2019).

4. Pedregosa, F. *et al.* Scikit-learn: Machine learning in Python. *J. Mach. Learn. Res.* **12**, 2825–2830 (2011).

5. Virtanen, P. *et al.* SciPy 1.0: Fundamental Algorithms for Scientific Computing in Python. *Nat. Methods* **17**, 261–272, DOI: 10.1038/s41592-019-0686-2 (2020).

6. Harris, C. R. *et al.* Array programming with NumPy. *Nature* **585**, 357–362, DOI: 10.1038/s41586-020-2649-2 (2020).
